# Acknowledgement to Reviewers of *Plants* in 2019

**DOI:** 10.3390/plants9010120

**Published:** 2020-01-17

**Authors:** Plants Editorial Office

**Affiliations:** MDPI, St. Alban-Anlage 66, 4052 Basel, Switzerland

The editorial team greatly appreciates the reviewers who have dedicated their considerable time and expertise to the journal’s rigorous editorial process over the past 12 months, regardless of whether the papers are finally published or not. In 2019, a total of 654 papers were published in the journal, with a median time to first decision of 14 days and a median time from submission to publication of 38 days. The editors would like to express their sincere gratitude to the following reviewers for their generous contribution in 2019:
Aalen, Reidunn BirgittaLiu, Shao-Lun (Allen)Abbate, LoredanaLiu, SijunAbdelrahman, MostafaLiu, WeizhenAbdel-Tawab, MonaLiu, YongliangAbe, AkiraLivanos, PantelisAbinaya, ManivannanLizamore, DarrellAbraham, EleniLlorens, EugenioAccoroni, StefanoLoake, GaryAchim, CristinaLognay, GeorgesÁcs, KamillaLogozzo, GiuseppinaÁdám, Attila L.Loizzo, Monica R.Adamakis, Ioannis-DimosthenisLoka, DimitraAdelberg, JefferyLomonaco, TommasoAdi, KliotLopes, GracilianaAgathokleous, EvgeniosLópez Saéz, José AntonioAgerbirk, NielsLoppi, StefanoAguiar, Francisca C.Lora, JorgeAhmed, MukhtarLord, JaniceAkhtar, YasminLordan, RonanAlam, Md. KhairulLorenz, PeterAlamillo, Josefa MuñozŁotocka, BarbaraAlba, ChristinaLøvdal, TrondAlbreht, AlenLoveridge, JoelAleksandrowicz-Trzcińska, MartaLovisolo, ClaudioAlexandrov, Oleg S.Lozano-Juste, JorgeAlexopoulos, AthanassiosLu, Chi-YuAl-Fatimi, MohamedLu, YouAlferez, FernandoLucas, Jose AntonioAlhousari, FadiLucek, KayAli, AkhtarLucena, CarlosAli Zaidi, ShanLucioli, SimonaAlison M., SmithLukhovitskaya, NinaAlkowni, RaedLullien, ValerieAllen, TomLunardon, AliceAltemimi, Ammar.BLutter, PetraAlvarez, Juan B.Lüttge, Ulrich E.Alves, SheilaLuxova, MiroslavaAlves, Tavvs MŁyczakowski, JanAmarowicz, RyszardMa, GuojiaAmes, Ryan M.Maag, DanielAmirkhani, MasoumeMabou Tagne, AlexAmsbury, SamMacfie, Sheila M.An, HongMaggi, FilippoAndolfi, AnnaMaghuly, FatemehAndrew, TedderMagnano Di San Lio, GaetanoAngelini, RiccardoMahdavi Mazdeh, ParastooAnikó, CsecseritsMaier, CameliaAnower, RokebulMaizel, AlexisAntal, DianaMalacrinò, AntoninoAntoniadi, IoannaMalla, SubasAntoniotti, SylvainMalladi, AnishAntoniou, ChrystallaMallory-Smith, CarolAntonkiewicz, JacekMalyan, SandeepAnver, ShajahanManfredini, StefanoAoki, KohMang, Stefania MirelaAparicio, FredericMano, Jun’ichiAppendino, GiovanniMantovani, RobertoAranda, IsmaelMantzouridou, FaniArcalis, ElsaMarasek-Ciolakowska, AgnieszkaArchMiller, Althea A.Marček, TihanaArena, CarmenMarcel, SylvainAres, ÁngelaMarchand, Patrice A.Arévalo, José RamónMarchi, MaurizioAroca, AngelesMarcon, CarolineArora, AmandeepMareri, LaviniaArturo Vera Ponce De Leon, ArturoMarhuenda, JavierArtyszak, ArkadiuszMarincas, OlivianAryal, RishiMarino, DanielAsard, HanMarkowicz-Piasecka, MagdalenaAscencio-Ibanez, TrinoMarra, RobertaAsquith, Christopher R. M.Marrelli, MariangelaAstier, JeremyMarsol, AlexisAstolfi, StefaniaMartí, RaúlAsztemborska, MonikaMartinez, ManuelAthanasios, DalakourasMartinez, ShantelAvellan, AstridMartínez, SidoniaAvesani, LindaMartinez Ruiz, CarolinaAvramidou, EvangeliaMartínez-Gómez, PedroAvramova, ViktoriyaMartínez-Sánchez, GregorioAxiotis, EvangelosMartínez-Turiño, SandraAzevedo, HerlanderMartino, ElenaAzzini, ElenaMartins, Alice M.Bacelar, EuniceMartins, IsabelBąchor, RemigiuszMartins-Loução, Maria AméliaBackor, MartinMartz, FrancoiseBadik, Kevin J.Maruta, TakanoriBaek, You SoonMaruyama-Nakashita, AkikoBagheri, HedayatMaserti, Bianca ElenaBagniewska-Zadworna, AgnieszkaMashima, RyuichiBahmani, RaminMasin, RobertaBai, HuaMasson, Patrick H.Bailly, ChristopheMastanjević, KrešimirBajaj, RuchikaMasuda, SachikoBaker, C. JacynMasuda, TatsuruBalasubramanian, BalamuralikrishnanMata-Pérez, CapillaBaldoni, ElenaMateo-Bonmatí, EduardoBalestrini, Raffaella MariaMatha, ShivarajBalslev, HenrikMathesius, UlrikeBan, YusukeMatichenkov, VladimirBanach, ArturMatilla, Angel J.Bandaralage, Jayeni Chathurika Amarathunga HitiMatilla, MiguelBanik, ChumkiMatsubara, KazukiBarberá, Gonzalo G.Matsumoto, TakahiroBarcelo, JuanMatthes, AnnemarieBaritaki, StavroulaMattoo, AutarBarnard-Kubow, KarenMattsson, JimBarreca, DavideMatuszewska, AnnaBarrios-Masias, Felipe H.Matzrafi, MaorBarrow, Chris J.Maulana, FrankBarta, CsengeleMayes, SeanBartosz, GrzegorzMayton, HilaryBaskin, Carol C.McFarlane, Heather E.Baslam, MarouaneMcInroy, John A.Bassi, DanieleMcintosh, RobertBassi, RobertoMcKenzie, Marian JaneBasu, SupratimMcKeown, Peter C.Baum, ChristelMcNeil, DavidBazghaleh, NavidMeans, NathanBazos, IoannisMedina, Francisco JavierBazylko, AgnieszkaMedo, JurajBazzicalupo, MarcoMeeks, CalvinBecana, ManuelMehariya, SanjeetBeck, AndreasMei, YangBeckie, Hugh J.Meisrimler, Claudia-NicoleBeers, EricMeki, NormanBegara-Morales, Juan C.Meletiou-Christou, Maria-SoniaBegcy, KevinMelosik, IwonaBehrends, VolkerMeneghetti, FiorellaBeilby, MaryMeneguzzo, FrancescoBeissinger, TimothyMenendez, EstherBellairs, SeanMenet, Marie ClaudeBellaloui, NacerMens, CelineBellucci, MicheleMensuali-Sodi, AnnaBenavides-Mendoza, AdalbertoMerah, OthmaneBencivenga, StefanoMerai, ZsuzsannaBender, JurgenMercati, FrancescoBender, KyleMerino, IreneBenfey, Philip NMészáros, IlonaBeng Kah, SongMeurer, JörgBenito, BegoñaMezzetti, BrunoBerberich, ThomasM-Hamvas, MártaBerendzen, Kenneth W.Miao, ChenyongBERGER, AntoineMichalak, MarcinBerger, SusanneMichaud, MorganeBergero, RobertaMichels, Alexander J.Berim, AnnaMierek-Adamska, AgnieszkaBerlanga, Ángel MéridaMilan, MarcoBerrocal-Lobo, MartaMinocha, SubhashBervillé, AndréMishra, Amit KumarBetekhtin, AlexanderMithoefer, AxelBetti, MarcoMittapelly, PriyankaBhusal, Siddhi J.Miyamoto, KojiBiagi, MarcoMizuno, TakayukiBiałoń, MariettaMogoșan, CristinaBiałowiec, AndrzejMohamed, Azza H.Bianco, ArmandodorianoMoir, JimBicknell, RossMolin, WilliamBidel, L.P.R.Molinier, JeanBiligetu, BillMøller, Ian MaxBilska-Kos, AnnaMonneveux, PhilippeBiniari, KaterinaMonteiro, FilipaBiondi, StefaniaMontes, NuriaBiselli, ChiaraMoolhuijzen, Paula M.Bishopp, AnthonyMoon, KyungBlanco, AntonioMoore, NathanBlanco-Salas, JoséMoraes, ThiagoBlaylock, Michael J.Moraga, JavierBleeker, Petra M.Morales, LauraBlein, ThomasMorales-Quintana, LuisBlein-Nicolas, MélisandeMorandi, BrunellaBoccacci, PaoloMoravcikova, JanaBocianowski, JanMorello, LauraBoczkowska, MajaMoreno, JordiBogdan, IleanaMorgan, JohnBohanec, BorutMori, KazuhiroBoisivon, Helene RobertMoricz, AgnesBoissinot, SylvaineMoriguchi, TakayaBölcskei, HedvigMorita, ShigetoBoldura, Oana-MariaMorra, Matthew J.Bolibok-Brągoszewska, HannaMorris, Paul FrancisBolle, CordeliaMorrow, JenniferBombarely, AurelianoMoschou, Panagiotis N.Bonciu, ElenaMota, ManuelBonello, PierluigiMottaghipisheh, JavadBonetta, DarioMotte, HansBonfil, DavidMourato, Miguel P.Borek, SławomirMucha, JoannaBoris, PejinMulas, MaurizioBoscaiu, MonicaMuleo, RosarioBostan, HamedMundy, D.C.Botton, AlessandroMuñoz, AlfonsoBoudsocq, MarieMuñoz-Centeno, Luz MaríaBouhaouel, ImenMurillo, Pablo GarcíaBouranis, Dimitris L.Mustroph, AngelikaBourque, StéphaneMuszyńska, EwaBouyahya, AbdelhakimMyrta, ArbenBoyce, RichardNadakuduti, Satya SwathiBozzo, Gale G.Nadeem, MuhammadBraam, JanetNadgórska-Socha, AleksandraBraglia, LucaNagahage, IsuraBrandt, KirstenNagashima, YukihiroBreidenbach, NatalieNagy, IstvanBresta, PanagiotaNakajima, MasamiBrestic, MarianNakamura, YukiBrew-Appiah, RhodaNakano, MichiharuBrewin, NickNakashima, SouichiBrighenti, VirginiaNallu, SumithaBrito, CátiaNamba, ShigetouBrogden, KimNapoli, MarcoBromfield, ElizabethNardi, SerenellaBrooks, PeterNardini, AndreaBrowse, JohnNardozza, SimonaBrukhin, VladimirNarouei-Khandan, HosseinBru-Martinez, RoqueNaumovski, NenadBrunetti, CeciliaNavarro-Alarcon, MiguelBrunkard, Jacob O.Nawade, BhagwatBrusslan, JudyNawaz, Muhammad AmjadBubici, GiovanniNeji, MohamedBuchholz, AnkeNelson, MatthewBudar, FrançoiseNemeskéri, EszterBueso, EduardoNeng, NunoBulgakov, VictorNeri, DavideBulli, PeterNeufeld, HowardBunce, JamesNeupane, AchalBurghardt, KyleNewcombe, GeorgeBurkey, KentNewman, Mari-AnneBurrows, Geoffrey E.Nicosia, AldoBusby, Ryan R.Niederman, Robert A.Butardo, VitoNielsen, BrentCabot, CatalinaNiemczyk, MarzenaCacciola, Santa OlgaNieto, GemaCádiz-Gurrea, María De La LuzNilsen, Erik T.Caeiro, MariaNin, StefaniaCaetano, NídiaNiu, GenhuaCahlíková, LucieNogales, AmaiaCahoon, A. BruceNoguchi, KoCai, GiampieroNogues, IsabelCalcutt, MichaelNonhebel, Heather M.Calha, IsabelNoordergraaf, Inge-WillemCalín-Sánchez, ÁngelNoris, EmanuelaCallahan, Damien L.Nosalewicz, ArturCallis, JudyNovikov, AndriyCalvano, Cosima DamianaNovikov, Arthur I.Calzarano, FrancescoNovo, LuísCamacho-Cristóbal, Juan J.Nürnberg, DennisCamañes, GemmaNybakken, LineCámara-Leret, RodrigoNybom, HildeCámara-Zapata, José MaríaO. Miller, JarrodCampbell, Malachy T.Ocamb, Cynthia M.Campbell, MichaelOfir, RivkaCampbell, ShaneOgata, YoshiyukiCañas, Rafael A.Ogórek, RafałCandido, VincenzoOgunjirin, AdebowaleCândido, ElizabeteOh, Seung-YoonCaneva, GiuliaOhtomo, RyoCanini, AntonellaOhtsu, NaokoCánovas, FranciscoOhyama, TakujiCantamessa, SimoneOkamura, YuCao, ZheOkazawa, AtsushiCapel, CarmenOklestkova, JanaCaputo, LuciaOkorski, AdamCaracciolo, GiuseppinaOkubara, PatriciaCaradonia, FedericaO'Leary, Brendan M.Cardarelli, MariateresaOlejar, Kenneth J.Cardinale, FrancescaOlmos, AntonioCardoso, HéliaOlmos, EnriqueCardoso, SusanaOlsson, SannaCarella, PhilipOmar, AhmadCarillo, PetroniaOmidbakhsh-Fard, M. AminCarocho, Márcio SoaresOndreičková, KatarínaCarol, PierreOndrej, VladanCarpin, SabineOng, Eng ShiCarrasco, PedroOniga, IlioaraCarroll, Charles J.W.Oniszczuk, AnnaCarrubba, AlessandraÖpik, MaarjaCarullo, GabrieleOrdas, BernardoCaruso, GianlucaOrlova-Bienkowskaja, Marina J.Carvajal, MicaelaO'Rourke, JamieCarvalho, Ana Maria PintoOrtega, JohnCasas, Ana M.Ortiz-Urquiza, AlmudenaCasciaro, BrunoOstrowski, MaciejCaser, MatteoOszako, TomaszCastilho, AlexandraOtagaki, ShungoCastilho, Paula C.Otulak-Kozieł, KatarzynaCastillo, JesúsOu, JunjunCastle, AlanOugham, HelenCastric, VincentOuzounidou, GeorgiaCastro, Pedro HumbertoOvermyer, KirkCastro-Vargas, Henry I.Owens, Daniel K.Cavaco, Ana M.Ozaki, NoriakiCazzato, EugenioOzimek, EwaCeliński, KonradPacuta, VladimirCelletti, SilviaPagán, IsraelČerný, MartinPagliarani, ChiaraCervantes, EmilioPagnotta, Mario A.Chagne, DavidPaixao Coelho, Jose AugustoChampion, CodyPająk, PaulinaChandrasekaran, MurugesanPál, MagdaChang, Wen-ChiPalacios, José-ManuelChang, Yu-SenPalacios, SaraChao, Louis KuopingPalanivelu, RaviChao, Wen-WanPalla, FrancoChapara, VenkatPalomo Ríos, ElenaChapman, MarkPalove Balang, PeterChardon, FabienPalumbo, FabioCharfeddine, SafaPanara, FrancescoChen, Jen-TsungPandolfini, TizianaChen, Po-YuanPanek, JacekChen, XunPapageorgiou, AristotelisChen, YinglongPapatheodorou, Efimia M.Cheng, Yuan-BinPapini, AlessioCheon, Choong-illPaponov, Ivan A.Cheung, AlicePappa, AglaiaChibbar, RavindraPappas, ChristosChin, Joong HyounParadjikovic, NadaChini, AndreaParajuli, SarojChitarra, WalterPark, ChankyuChiurazzi, MaurizioPark, Il-KwonChlichlia, KaterinaPark, InkyuChmara, RafałPark, Sang-WookChmura, DamianPark, SunghunCho, Man HoParker, Steven L.Chorostecki, UcielPârvu, Alina ElenaChristoffers, MichaelPasternak, TarasChrysargyris, AntoniosPatil, AbhinandanChrysoula, TananakiPaul, MatthewChun, SechulPaul, Narayan ChandraCilas, ChristianPavela, RomanCillo, FabrizioPazzagli, LuigiaCirvilleri, GabriellaPečenková, TamaraClark, GaryPedersen, HenrikClark, GregoryPedrazzini, EmanuelaCober, ElroyPedroza-Garcia, Jose-AntonioCocozza, ClaudiaPeirats-Llobet, MartaCoelho, LiviaPeixe, AugustoCogato, AlessiaPék, ZoltánCohen, Yigal R.Peltomaa, ElinaCole, Logan W.Peña-Gómez, NatalyCollet, TrudiPeng, ZeCollino, MassimoPerdomo, Juan AlejandroCombier, Jean-philippePereira, Paula NatáliaCominelli, EleonoraPereira-Lorenzo, SantiagoCommisso, MauroPerez, MartaConti, HeatherPerez De Souza, LeonardoConway, WarrenPérez-Fernández, MaríaCooke, JuliaPernisová, MarkétaCoppi, AndreaPernot, ClémentineCordeiro, NereidaPerotto, SilviaCorpas, JavierPerrine-Walker, FrancineCorreia, Carlos M.Perry, SharynCorreia, PedroPerucka, IrenaCosoveanu, AndreeaPeška, VratislavCosta, FabrizioPetek, MarkoCostello, Ben De LacyPetoumenou, Despoina G.Cox, RobertPetricevich, VeraCozzolino, DanielPetrivalsky, MarekCretu, MirelaPetropoulos, SpyridonCrevillén, PedroPetrussa, ElisaCrișan, GianinaPetti, CarloalbertoCrisp, PeterPeyambari, MahtabCruz, CristinaPfister, BarbaraCséplő, ÁgnesPiasecki, CristianoCvrčková, HelenaPiccionello, Antonio PalumboCzerwinska, MonikaPiccolella, SimonaCzerwińska, MonikaPickard, BarbaraDa Silva, Jorge MarquesPiechulla, BirgitDagnon, SoleyaPiedras, PedroD'Agostino, NunzioPiemontese, LucaDal Santo, SilviaPiepenbring, MeikeDamalas, Christos A.Piepho, Hans-PeterDANG, THU-THUYPietrowska-Borek, MałgorzataDanso-Boateng, EricPiispanen, RiikkaDaras, GerasimosPilu, RobertoDasgupta, KasturiPingault, LiseDatta, RahulPinker, InaDávalos, AndreaPitingolo, GabrieleDavid, KopeckyPiya, SarbottamDavid, LaurePizzeghello, DiegoDavid, LuminitaPlader, WojciechDavies, Peter J.Platta, HaraldDe Andrade, AlexanderPŁażek, AgnieszkaDe Bellis, LuigiPleskot, RomanDe Diego, NuriaPlíhal, OndřejDe Graaf, Barend HJPlíhalová, LucieDe Masi, LuigiPniewski, TomaszDe Mieri, MariaPokluda, RobertDe Paolis, AngeloPolidoros, AlexiosDe Tullio, Mario C.Pollastro, StefaniaDe Vendittis, EmmanuelePongrac, PaulaDe VIENNE, DominiquePonte, NunoDe Vries, JanPonz, FernandoDebnath, SamirPoór, PéterDeckert, JoannaPopova, AntoanetaDehghan, EsmaeilPorceddu, MarcoDel Genio, CharoPotrykus, Martadel Pozo, J. CarlosPoudyal, ShitalDel Río Celestino, MercedesPourret, OlivierDelgado, Juan AntonioPozzi, CeciliaDelgado, Sergio NogalesPrado-Cabrero, AlfonsoDello Ioio, RaffaelePramod, SreepriyaDenholm, IanPranckeviciene, ErinijaDeram, AnnabellePrasad, Kasavajhala V. S. K.Dermastia, MarinaPrasad, RamDeshmukh, RupeshPrashar, SanjivDevireddy, AmithPremachandra, Gnanasiri S.Devlin, PaulPretel, Maria TeresaDhandapani, VigneshPrice, Elliott JamesDharmasiri, NihalPringle, ElizabethDhiman, SaurabhPrinsen, ElsDi Bene, ClaudiaPrudent, MarionDi Gioia, FrancescoPucci, NicolettaDi Mambro, RiccardoPuccinelli, MartinaDi Martino, CatelloPucker, BoasDi Mola, IdaPuglia, GiuseppeDi Rita, FedericoPugliesi, ClaudioDi Sansebastiano, Gian-PietroPuglisi, IvanaDiaba, FaïzaPugovics, OsvaldsDietzgen, RalfPuig, Carolina G.DiGennaro, PeterQaderi, MirwaisDijk, AaltQiu, HuanDimitrakopoulos, Panayiotis G.Quambusch, MonaDing, BaoqingQuerner, PascalDing, JingyiQuesada Pérez, VíctorDinis, Lia Tânia JRace, MarcoDinkova, Tzvetanka D.Radford, Ian J.Diogène, JorgeRadić, TomislavDivashuk, MikhailRahi, LifatDjalali Farahani-Kofoet, RoxanaRahman, Md. AtikurDjaman, KoffiRaikhy, GauravDobrikova, AneliaRajarapu, Swapna PriyaDocimo, TeresaRallo, LuisDodd, AntonyRamana Pidatala, VenkataDodd, IanRamegowda, VenkategowdaDoddapattar, PrakashRamesh, Sunita A.Doi, KazuyukiRamos-Solano, BeatrizDolatabadian, AriaRapeanu, GabrielaDombrovsky, AvivRascio, AgataDomozych, David S.Rasmussen, SorenDong, YiboRass, UlrichDonno, DarioRastogi, AnshuDoria, EnricoRatcliffe, Richard GeorgeDos Reis, André RodriguesRaudabaugh, DanielDOSOKY, NOURARaudonė, LinaDrabińska, NataliaRavelonandro, MichelDrucker, MartinRavin, Nikolai V.Duan, JialeiRavlić, MarijaDuarte, Maria CristinaRayon, CatherineDubreucq, BertrandReda, MałgorzataDubrovina, Alexandra S.Reddy Janga, MadhusudanaDugé De Bernonville, ThomasReed, Kevin F.M.Dun, ElizabethReglinski, TonyDuraisamy, Ganesh SelvarajRegni, LucaDurgo, KsenijaRehman, Junaid UrDyderski, MarcinRehrig, Erin M.Dyer, WilliamReig, GemmaDziadas, MariuszReigosa, Roger J.Eamens, Andrew L.Ren, HongEbert, Timothy ARenault, SylvieEckerstorfer, MichaelRenna, MassimilianoEdqvist, JohanResentini, FrancescaEdwards, JeremyRestuccia, AlessiaEgamberdieva, DilfuzaRevuelta, JuliaEgerton-Warburton, LouiseRezzani, RitaEibl, RegineRho, HyungminElameen, AbdelhameedRial, CarlosElansary, Hosam O.Ricci, AntonellaEleftheriou, Eleftherios P.Richard, BenjaminEl-Esawi, MohamedRichter, RenéElkins, KellyRigas, StamatisElshamy, AbdelsamedRigó, GáborEndresen, DagRigoldi, Maria PiaEngelberth, JurgenRischer, HeikoErel, RanRivero, Rosa M.Erktan, AmandineRoach, ThomasErmilova, ElenaRobert, Elisabeth M. R.Ersoz, Elhan S.Robin, Arif Hasan KhanErtani, AndreaRoche, BenjaminEzer, DaphneRoden, JohnFalade, Titilayo D.O.Rodrigo, JoaquinFalavigna, VitorRodrigues, António S.Falistocco, EgiziaRodrigues, Célia F.Fambrini, MarcoRodríguez, Víctor ManuelFang, FrancisRodríguez De Rivera, ÓscarFanizzi, Francesco PaoloRodríguez Villanueva, JavierFanourakis, DimitriosRodríguez-González, ÁlvaroFaraone, ImmacolataRodríguez-Merino, ArgantonioFarcas, Anca CorinaRodríguez-Peña, Rosa A.Fares, ClaraRodríguez-Soalleiro, RoqueFarzaneh, VahidRodríguez-Yoldi, María JesúsFascella, GiancarloRogers, HilaryFasoula, VasiliaRogozhin, EugeneFedderwitz, FraukeRohila, JaiFehér, AttilaRollin, PatrickFekete, AgnesRomeiras, Maria M.Felippes, Felipe Fenselau DeRomeiras, Maria ManuelFeller, UrsRomero, IreneFenesi, AnnamáriaRomero, JoséFernández, José A.Romero-Puertas, María C.Fernández Barbero, GerardoRomuli, SebastianFerrante, ClaudioRonga, DomenicoFerrenberg, ScottRoni, Md Zohurul KadirFerrie, A. M. R.Ros, RocFerroni, LorenzoRoscini, LucaFestuccia, NicolaRosellini, DanieleFetcher, NedRossato, MarziaFialová, SilviaRoubelakis-Angelakis, Kalliopi A.Figaj, DonataRoutray, WinnyFigueroa-Bustos, VictoriaRoyo-Esnal, AritzFikere, MulusewRoyuela, MercedesFilip, LorenaRozhon, WilfriedFischer-Schrader, KatrinRozov, Serge M.Flakus, AdamRóżyło, KrzysztofFlores García, EnriqueRudnóy, SzabolcsFlorez-Sarasa, IgorRudrabhatla, SairamFoereid, BenteRudzińska, MagdalenaFormenti, LudovicoRuelland, EricForrer, Hans RudolfRuggeri, RobertoForster, BrittaRugini, EddoForti, LucaRuiz Talonia, LorenaFotopoulos, VasileiosRuiz Téllez, TrinidadFrancioso, OrnellaRunions, AdamFranssen, HenkRurek, MichalFrolov, AndrejRussell, ScottFrugis, GiovannaRusso, DanielaFu, Yong-BiRutto, LabanFujii, HiroakiRybczyńska-Tkaczyk, KamilaFujii, ShoRybka, KrystynaFujimoto, RyoSaarto, AnnikaFujita, DaisukeSabater, BartolomeFujita, MasayukiSabella, ErikaFujiwara, TakayukiSadot, EinatFujiyama, HideyasuSaha, Sushanta KumarFukuda, TomohikoSahab, SareenaFukui, KenjiSahrawy, MariamFukunaga, KenjiSaiano, FilippoFukushima, AtsushiSaitanis, CostantinosFukushima, KenjiSaito, YoshiroFunada, RyoSakamoto, TomoakiFung Uceda, JorgeSakar, GermánFunnekotter, BrynSakata, YuzuGabbia, DanielaSakkas, HerculesGabrielli, PaoloSalachna, PiotrGabryś, BeataSalas, José BlancoGaggini, LucaSalmerón-Sánchez, EstebanGalasiti Kankanamalage, AnushkaSalome, PatriceGaliba, GaborSalopek-Sondi, BrankaGallardo-Guerrero, KarineSamach, AlonGallego, Juan Carlos AlíasŠamec, DunjaGallemí, MarçalSanchez, DarleneGaloburda, RutaSánchez Torres, PalomaGalstyan, AnahitSandalio, Luisa MaríaGalvão, Vinicius CostaSangireddy, Sasikiran ReddyGambino, GiorgioSansone, AnnaGan, SushengSantino, AngeloGanesan, PalanivelSantos, Ana PaulaGangola, Manu PratapSantos, Bruno A C MGanič, Karin KovačevićSantos, Carla S.Gannon, BryanSantos, Rita B.Garcia De Leon, DavidSantos, SusanaGarcía-Cervigón, Ana I.Sanz-Alférez, SoledadGarcía-Estañ, JoaquínSanz-Saez, AlvaroGarcia-Sanchez, FranciscoSara, MontanariGarmendia, IdoiaSarkar, AbhijitGarzoli, StefaniaSarkar, Saswata SankarGasparatos, DionisiosSatish, LakkakulaGasperini, DeboraSato, HikaruGates, ColinSato, NorihiroGatz, ChristianeSattar, SampurnaGauslaa, YngvarSauer, MichaelGavilan, RosarioSavchenko, Tatyana V.Gavilán, Rosario G.Savoi, StefaniaGeisler, MattSawicki, RafalGent, MartinScala, ValeriaGenta-Jouve, GrégoryScalabrin, ElisaGeorgiev, VasilScandellari, FrancescaGeorgiev, YordanScarafoni, AlessioGerke, JörgScavo, AurelioGerm, MatejaSchaffer, Arthur A.Gervasi, TeresaSchaller, JörgGesell, AndreasSchaub, MarcusGhanizadeh, HosseinSchiavon, MichelaGhatak, ArindamSchlüter, UrteGhosal, SambuddhaSchmeda-Hirschmann, GuillermoGhose, KaushikSchmidt, LilianGiacomello, StefaniaSchmidt, MartinGianinetti, AlbertoSchmitt-Keichinger, CorinneGibson, David JohnSchoenaers, SébastjenGilardi, GiovannaSchreier, TinaGilding, Edward K.Schroeder, HilkeGiles, WayneSchubert, MarianGiordani, PaoloSchulz, AlexanderGiordano, SimonettaSchulze, StefanieGiritch, AnatoliSchuster, GadiGiurg, MirosławSchuster, MichaelGladish, Daniel K.Scognamiglio, MonicaGlibowski, PawełScorziello, AntonellaGłowacka, KatarzynaScotti, NunziaGodena, SaraSebastiana, MónicaGoicoechea, NievesSebastiani, FedericoGoldfarb, BarrySeca, Ana Maria Loureiro DaGoldschmidt, Eliezer E.Segura, AnaGollan, Peter J.Seifert, Georg J.Golob, AleksandraSeo, EunyoungGolubkina, NadezhdaSerafini, MauroGomes, EricSerafini-Fracasini, DanatellaGómez Cadenas, AurelioSERALINI, Gilles-EricGomez Camacho, Carlos EnriqueSergeant, KjellGomez Roldan, Maria VictoriaSerrano, MaríaGonçalves, SandraSetamou, MamoudouGonda, SandorSeto, ShigekiGontier, EricSetzer, William N.González Andújar, José LuisSeung, DavidGonzález-Domínguez, RaúlSevilla-Morán, BeatrizGorchov, DavidSgorbini, BarbaraGori, AntonellaShabbir, AsadGorres, Josef H.Shafiq, SarfrazGospodarek, JaninaShahrokhnia, HosseinGossman, ToniShahzad, BabarGoyal, EtikaShaikhali, JehadGrabelnych, Olga I.Sharma, HarmandeepGrace, JohnSharma, ManishaGradov, Oleg V.Sharpe, Richard M.Gradziel, Thomas M.Shen, JianqiangGrafi, GideonSheng, RuilongGramzow, LydiaShi, XiaowenGranica, SebastianShibuya, KenichiGrantz, DavidShim, DonghwanGrattan, StephenShimada, TomooGreenberg, AnthonyShimojima, MieGreenlief, C. MichaelShiratani, MasaharuGreer, DennisShishova, Maria F.Gregersen, PerShrestha, KrishnaGrey, TimShukla, MukundGrones, PeterShymanovich, TatsianaGrote, RüdigerSiatkowski, IdziGroten, KarinSidhu, GaganjotGrubišić, DinkaSidhu, VirinderGrul’ová, DanielaSieber, Alisa-NaomiGrumet, RebeccaSilar, PhilippeGrzebelus, DariuszŠiler, BranislavGrzebelus, EwaSilva Dias, LuísGu, XingyouSilvestri, CristianGuadagno, CarmelaSimkin, AndrewGueguen, ErwanSimm, StefanGuerra-Sanchez, GuadalupeSims, BrianGuerriero, GeaSingh, AshutoshGuevara, RamónSingh, ManjulGuevara González, RamónSingh, NarinderGuil-Guerrero, José LuisSingh, ReemaGujas, BojanSipes, BrentGullì, MariolinaSivanesan, IyyakkannuGuo, Hao-BoSkaracis, GeorgeGuo, ZhanjunSkendi, AdrianaGupta, ParulSkjelvåg, Arne OddvarGutekunst, KirstinSkorko-Glonek, JoannaGutiérrez-Miceli, FedericoSkrypnik, Liubov N.Guy, Charles L.Ślipiko, MonikaGuzmán-Delgado, PaulaŚliwińska-Wilczewska, SylwiaH. Gonzalez, DanielSloan, DanielHa, Chien VanSlominski, AndrzejHădărugă, NicoletaŚlusarczyk, SylwesterHagerty, ChristinaSmedley, Mark A.Halassy, MelindaSmeriglio, AntonellaHamman, Sarah T.Smetana, OndrejHammerschmidt, RaySmith, Mark A.Hammond, JohnSng, Natasha J.Hamouzová, KateřinaSoares, CristianoHan, Yeon SooSobieszczuk-Nowicka, EwaHanaka, AgnieszkaSogawa, ChiharuHanania, MichelSolanki, Manoj KumarHanaoka, MitsumasaSolano, FranciscoHancock, JohnSoldatenko, Sergei A.Hano, ChristopheSolovyev, NikolayHanus-Fajerska, EwaSoltabayeva, AigerimHardion, LaurentSołtys-Kalina, DorotaHarman, GarySommaggio, DanieleHarmens, HarrySong, GuoqingHarrington, Kerry C.Soriano, Jose MiguelHartl, MarkusSosnowska, KatarzynaHasanuzzaman, MirzaSousa, Joana L. C.Haseeb, MuhammadSouth, Paul F.Hasegawa, Paul MichaelSouza, PedroHasiów-Jaroszewska, BeataSowa, IreneuszHassan, Sherif T. S.Spałek, KrzysztofHatano, TsutomuŠpanič, ValentinaHatzpolous, PolydefkisSpeciale, AntonioHause, BettinaSpiegel, HolgerHawrylak-Nowak, BarbaraSpyroglou, GavriilHay, AngelaSreedasyam, AvinashHayashi, SatomiSroka, ZbigniewHayes, PatrickStacewicz-Sapuntzakis, MariaHearn, DavidStadler, MarcHeckenhauer, JacquelineStankovic, MilanHedden, PeterStaples, TimothyHeger, TinaStarfinger, UweHell, RüdigerStarkuviene, VytauteHelliwell, ChrisStarns, HeathHelm, RichardStarowicz, MałgorzataHenry, RobertStefanaki, AnastasiaHerath, VenuraStefanou, StefanosHernández, José A.Stelinski, Lukasz L.Herranz, RaúlStevanato, PiergiorgioHerrero, BaudilioStevens, Rebecca G.Herzberg, MartinStewart Jr., CharlesHess, MichaelStobiecki, MaciejHessel-Pras, StefanieStoleru, VasileHeyer, ArndStotz, Henrik U.Higgs, DavidStout, Michael J.Hilliou, FrederiqueStrati, Irini F.Hinojosa, LeonardoStuart, JeffHirakawa, YoshihisaStudnicki, MarcinHirel, BertrandStührwohldt, NilsHirotsu, NaokiStuper-Szablewska, KingaHirschi, KendalStützel, HartmutHo, HonSubramani, MayavanHodson, MartinSubramani Paranthaman, BalasubramaniHojsgaard, DiegoSuen, Der-FenHolalu, SrinidhiSugier, PiotrHolec, JosefSullivan, StuartHölzl, GeorgSun, FengjieHong, YiguoSung, JwakyungHorel, ÁgotaSung, Shi-Jean S.Hori, KiyosumiSung, SibumHoriguchi, GorouSurapathrudu, KanakalaHortigón-Vinagre, MaríaSuryawanshi, VasantikaHorvath, DavidSuwazono, YasushiHossein, BorhanSuzaki, TakuyaHosseini, Seyed AbdollahSuzuki, NobuhiroHowe, GreggSuzuki, YoshihitoHsia, Shih-MinSwiezewska, EwaHsieh, Hsu-LiangSword Sayer, Mary AnneHsu, Yi TingSynowiec, AgnieszkaHu, GuanjingSyromyatnikov, Mikhail Y.Huang, Guan-JhongSytar, OksanaHuang, LeiSzczuka, EwaHuang, ShuanglongSzepesi, AgnesHuang, Wen-LiiSzewczyk, KatarzynaHuang, XingfengSzumny, AntoniHubert, SytykiewiczSzwengiel, ArturHuchzermeyer, BernhardSzydlowski, NicolasHura, TomaszSzymanowska, UrszulaHusbands, AmanSzymanowska-Pułka, JoannaHwang, Woon-HaTabassum, SamiyaIan, DundasTacchini, MassimoIanole, RodicaTada, YuichiIantcheva, AneliaTaguchi-Shiobara, FumioIapichino, GiovanniTakabayashi, JunjiIbba, Maria ItriaTakada, YoshinobuIbrahim, SalamTakagi, DaisukeIentile, RiccardoTakahashi, MisaIgamberdiev, Abir U.Takahashi, ToshiyukiIkeda, HajimeTakano, AtsukoIlardi, VincenzaTakeda, SeijiImamura, SousukeTamaoki, MasanoriInagaki, NoritoshiTanaka, HisashiInnangi, MicheleTang, RenjieIorizzi, MariaTang, YuhongIrga, PeterTaranto, FrancescaIrish, ErinTarkowská, DanušeIrving, LouisTarkowski, PetrIshiguro, SumieTate, Rothwelle J.Ishijima, SumioTattini, MassimilianoIshikawa, RyujiTava, AldoIshizaki, TakumaTeeri, TeemuIshizuka, WataruTeh, Soon LiIslam, Md. TabibulTeixeira, RitaIslam, Mohammad ZahirulTekiela, DanielItabashi, EtsukoTelewski, FrankIto, MichihoTeresa, Del Castillo-SantaellaIvanov, AlexanderTerol, JavierIvanov, IvanTerpolilli, Jason JIwakami, SatoshiTervahauta, Arja IIwata, YujiTerzaghi, WilliamJ. Barba, FranciscoTétard-Jones, CatherineJabloun, MohamedTetlow, IanJain, Shri MohanThalmann, MatthiasJakkula, VinodThammina, Chandra SekharJallet, DenisThérèse Charles, MarieJan, Fuh-JyhThilmony, RogerJanda, ElzbietaThiruvengadam, MuthuJang, SeonghoeThomas, FrankJanga, MadhusudhanaThomson, LindaJanicka-Russak, MałgorzataThorwarth, PatrickJanssen, DirkTian, LiJaradat, NidalTielbörger, KatjaJarolmasjed, SanazTo, Kin-YingJarquin, DiegoTobias, PeriJarret, Robert L.Todaka, DaisukeJarugula, SridharTodorova, Dessislava A.Jeandroz, SylvainTokarska-Guzik, BarbaraJellen, EricTokuhisa, JamesJeong, Dong-HoonTomczyk, MichalJeong, Rae-DongTomczykowa, MonikaJerkowić, IgorTomi, FélixJewell, Jeremy B.Tomkowiak, AgnieszkaJhanwar, ShaluTomooka, NorihikoJiang, Shu-YeTopalidou, EleniJicsinszky, LászlóToriba, TaiyoJiménez Fernández, AliciaTorn, KaireJimenez-Lopez, Jose CarlosTorra, JoelJobe, TimothyTorricelli, RenzoJocković, MilanToyota, KoikiJohnson, DonnTrammell, SamJohnson, EricTranbarger, Timothy JohnJohnson, GilesTraw, Milton BrianJones, MerielTrcek, JanjaJones, Richard WTrdan, StanislavJopcik, MartinTreder, JadwigaJordan-Meille, LionelTrentini, AlessandroJordão, António ManuelTripodi, GianlucaJordheim, MonicaTroitsky, AlexeyJørgensen, Hans Jørgen LyngsTrotta, AndreaJorrin, BeatrizTrovato, MaurizioJose, RodriguezTrumble, JohnJuárez-Escario, AlejandroTsakirpaloglou, NikolaosJulien, GronnierTsaltas, DimitrisJullian, NathalieTseng, Chih-HuaJung, Ki-HongTsitsekian, DikranJuráň, StanislavTubana, BrendaJurkonienė, SigitaTuenter, EmmyKadela-Tomanek, MonikaTuncel, AytugKafle, LekhnathTundis, RosaKAINOH, YooichiTuomivirta, TeroKaitaniemi, PekkaTurkovskaya, Olga V.Kakrana, AtulTurner, ShaneKalachova, TetianaTwardowski, TomaszKalaji, Hazem MohamedTyagi, SwatiKalemba, Ewa M.Tyrka, MirosławKaliniewicz, ZdzisławUeda, MinoruKalyuzhnaya, MarinaUesugi, AkaneKaminaka, HironoriUimari, AnneKanehara, KazueUllah, ChhanaKanissery, Ramdas G.Umehara, MikihisaKapantaidaki, Despoina Ev.Upadhyay, Rakesh K.Karahara, IchirouUprety, YadavKarcz, DariuszUrbaniak, JacekKarkanis, AnestisUrza, Alexandra K.Karmakar, ParthaUygun, SahraKaroglan, MarkoValentine, TracyKartashov, AlexanderValenzuela, Juan LuisKasahara, Ryushiro D.Valkov, VladimirKato, KeitaValletta, AlessioKato, YoshioVallini, GiovanniKatsuhara, MakiVan den Berg, CássioKaur, HarmanjitVan Der Linde, KarinaKawade, KensukeVan Klink, John WilliamKawamura, FumioVan Os, ErikKayama, MasazumiVan Velzen, Ewoud J. J.Kazama, TomohikoVan Wuytswinkel, OlivierKeereetaweep, JantanaVarallyay, EvaKeinänen, MarkkuVaroni, Elena MariaKellogg, Joshua J.Varotto, SerenaKenter, ChristineVaughan, Martha M.Kern, RamonaVeatch-Blohm, MEKerwin, Rachel E.Vencill, WilliamKerwin, Sean M.Venkatesan, ThiagarajanKhallouki, FaridVenturi, FrancescaKhanal, SameerVenugopal, MenduKhazaei, HamidVeres, SzilviaKheradpezhouh, EhsanVerger, StéphaneKhosla, AashimaVergine, MarziaKibet, LeonardVerstraeten, IngeKikowska, MałgorzataVicario, SaverioKil, Eui-JoonVicente, Joana G.Kim, In-JungVicente Pérez, RubénKim, Jin-HongVideau, PatrickKim, Jin-KyungVielba, Jesús MaríaKim, Ki HyunVigani, GianpieroKim, Kyung-MinVillegas, DolorsKim, SukweonVincenot, Christian ErnestKim, Tae-HwanVincourt, PatrickKim, Woe-YeonVining, KellyKim, Yong-IckVinyard, DavidKimbaris, AthanasiosVirdi, KamaldeepKimura, SeisukeVirjamo, VirpiKinose, YoshiyukiVítová, MiladaKinugasa, ToshihikoVitti, AntonellaKiraly, LorantVittorioso, PaolaKiselev, Konstantin V.Vodnar, Dan CristianKlauser, DominikVojta, LeaKlčová, LenkaVolaire, FlorenceKleczkowski, Leszek A.Volkov, VadimKnoshaug, EricVurro, MaurizioKo, KisungVuts, JozsefKobae, YoshihiroVyhnánek, TomášKocábek, TomášWajs-Bonikowska, AnnaKocira, SławomirWalker, Robert J.Koga, HiroyukiWanasundara, JanithaKoide, YoheiWang, Chao-MinKoizuka, NobuyaWang, Chih-LiKoji, MikamiWang, JinxiangKolbert, ZsuzsannaWang, PengKollárová, KarinWang, Sheng-YangKolodziejczyk, JoannaWang, SishuoKolukisaoglu, ÜnerWang, XutongKomenda, JosefWang, YifeiKomínková, DanaWang, YingKomis, GeorgeWang, YuanKomis, GeorgeWang, ZhenglinKonar, ArkaprabhaWard, JohnKong Thoo Lin, PaulWarner, DouglasKonopka, IwonaWaszczak, CezaryKonopka-Postupolska, DorotaWatanabe, MakotoKontunen-Soppela, SariWei, RobertKooij, Pepijn W.Weiss, JuliaKorell, LotteWen, Amy M.Kosmala, MonikaWeng, Mao LunKosova, KlaraWeng, XiaoyuKosterin, OlegWeraduwage, Sarathi M.Kotta-Loizou, IolyWest, Natalie MKowalczyk, MariuszWhitney, CoryKowalski, RadosławWidemann, EmilieKozaki, AkikoWiersma, Andrew T.Kozieł, EdmundWiggins, GregKozlov, Mikhail V.Wilkinson, Mark D.Kraj, WojciechWillick, IanKramer, Elena M.Wilmowicz, EmiliaKrasniewska, KarolinaWimmer, Monika A.Kriauciuniene, ZitaWinkler, Daniel E.Kriechbaumer, VerenaWinkworth, RichardKrier, FrançoisWinterhagen, PatrickKrishnamurthy, PannagaWishart, JaneKroj, ThomasWiszniewska, AlinaKrüger, StephanWitcher, AnthonyKuboyama, TsutomuWitte, Claus-PeterKucukoglu, MelisWittkop, BenjaminKudoyarova, GuzelWojtunik-Kulesza, Karolina A.Kukal, MeetpalWojtyla, ŁukaszKulhánek, MartinWolny, ElzbietaKulich, IvanWoodrow, PasqualinaKulkarni, ShashankWoodward, AndrewKulma, AnnaWoznicki, Tomasz L.Kulus, DariuszWu, HaoKumar, JitendraWu, HonghongKumar, ManuWu, ShanKumar, NarenderWullschleger, Stan D.Kumar Kundu, JibanWyszkowska, JadwigaKunihisa, MiyukiXavier, AlencarKunwar, Ripu MardhanXiao, WenyanKuo, Yao-HaurXu, PengKuta, ElżbietaXu, YiKwiatkowski, PawelXuan, Tran DangKwon, Choon-TakYahata, MasakiKwon, Suk-YoonYahr, RebeccaLa Spada, FedericoYamagata, YoshiyukiLaane, Henk-MaartenYamakawa, TakeoLabate, JoanneYamasaki, HideoLabudda, MateuszYan, MaryLachman, JaromirYandeau-Nelson, Marna D.Lacis, GunarsYang, GaowenLahuta, Lesław BernardYang, PeiqiLam, Sio-HongYang, Seung HwanLamb, Dane T.Yang, Shun-ChinLandoni, MichelaYang, Yu-ChiaoLange, BastienYe, LiyunLangner, ThorstenYeh, Ching-HuiLao Arenas, Maria TeresaYeung, ElaineLaranjo, MartaYing, ShengLarkan, NicholasYokosyo, KengoLarkum, AnthonyYokota, ShinsoLarson, Steven R.Yoshida, Kaoru T.Lastochkina, OksanaYoshida, KeisukeLászló, MakraYou, ChenjiangLatowski, DariuszYou, Frank M.Laur, JoanYu, XiangLavelle, Dean O.Yu, XingwangLavelli, VeraYuann, Jeu-Ming P.Lavin, MattZabetakis, IoannisLazarević, BorisZacchini, MassimoLazaro, LaurenZahoranova, AnnaLe Hir, RozennZaidi, Syed Shan-E-AliLebedev, Vadim G.Zaini, PauloLeborgne-Castel, NathalieZaitlin, DavidLedeunff, ErwanZakrzewski, JerzyLee, Byung-HooZamboni, AnitaLee, Jung RoZamyatnin, AndreyLee, KeunsubZancani, MarcoLee, NatuschkaZapata Coll, Pedro JavierLehtonen, SamuliZapata-Sierra, AntonioLeitão, José M.Zarabska-Bożejewicz, DariaLeiva-Brondo, MiguelZarrelli, ArmandoLemos, Rafael Plá MatieloZarrouk, OlfaLentini, GiovanniZell, RolandLeon-González, Antonio J.Zeller, Alexander K.León-Rivera, IsmaelZhai, ZhiyangLepeduš, HrvojeZhang, CankuiLeru, PolianaZhang, FeiLeszczuk, AgataZhang, JinLevitsky, Victor G.Zhang, JunliLeyva Pérez, Maria De La OZhang, WeiLi, Po-HsienZhang, WenhengLi, QianshengZhao, ChengLi, WanlongZhao, DongyanLi, XiaoguangZhao, JianfeiLi, YinZhao, JinpingLi, ZhongyiZhou, BangjunLi Destri Nicosia, Maria GiuliaZhou, LinLiLiang, ZhikaiZhou, RongLiao, YuxingZhou, ZhenjiangLico, ChiaraZhu, ChangfuLidon, FernandoZhu, YingjieLigaba-Osena, AyalewŽiarovská, JanaLiguori, GiorgiaZielińska, SylwiaLima-Brito, JoséZimmermann, Sabine DagmarLin, Hung-YinZiogas, VasileiosLin, Tung-YiZitko, JanLin, Wei-YiZivcak, MarekLing, MauriceZovko Končić, MarijanaLinhari Rodrigues Pietro, Rosemeire CristinaŹróbek-Sokolnik, AnnaLinkies, AdaZub, KarolLionetti, VincenzoZubieta, ChloeLiu, HaipeiZuk, MagdalenaLiu, KeZuluaga, PaolaLiu, LeiŻurek, GrzegorzLiu, LiyunZuriaga, ElenaLiu, Ming-JungZyprian, EvaLiu, Qing


